# A Comparison Study on the Strengthening and Toughening Mechanism between Cu-Bearing Age-Hardening Steel and NiCrMoV Steel

**DOI:** 10.3390/ma14154276

**Published:** 2021-07-30

**Authors:** Xiaobing Luo, Chongchen Xiang, Feng Chai, Zijian Wang, Zhengyan Zhang, Hanlin Ding

**Affiliations:** 1 Department of Structural Steels, Central Iron and Steel Research Institute, Beijing 100081, China; luosir2007@sina.com (X.L.); zhangzhengyan@cisri.com.cn (Z.Z.); 2 School of Iron and Steel, Soochow University, Suzhou 215006, China; ccxiang@suda.edu.cn (C.X.); wangzijian@suda.edu.cn (Z.W.)

**Keywords:** Cu-bearing age-hardening steel, precipitation, Cu-rich particles, mechanical properties

## Abstract

Cu-bearing age-hardening steel has significant potential in shipbuilding applications due to its excellent weldability as compared to conventional NiCrMoV steel. Not much research has been carried out to analyze the differences in the mechanisms of strength and toughness between Cu-bearing age-hardening and NiCrMoV steel. Both steels were heat treated under the same conditions: they were austenized at 900 °C and then quenched to room temperature, followed by tempering at 630 °C for 2 h. The uniaxial tensile test reveals that the Cu-bearing age-hardening steel exhibits relatively lower strength but larger plasticity than NiCrMoV steel. The lower contents of Carbon and other alloying elements is one of possible reasons for these differences in mechanical properties. Transmission Electron Microscope observations show that two types of precipitates, Cr carbides and Cu-rich particles, exist in tempered Cu-bearing age-hardening steel. Cu-rich particles with sizes of 20–40 nm can inhibit the dislocation motion during deformation, which then results in dislocation pile ups and multiplication; this makes up the strength loss of Cu-bearing age-hardening steel and simultaneously improves its plasticity.

## 1. Introduction

The development of shipbuilding steels has gone through a process from carbon steel to low-carbon low-alloy steel, and further to ultra-low-carbon low-alloy steel [1,2]. The primary reason for this tendency is that the weldability of shipbuilding steels decreases dramatically with the increasing carbon content and the untempered martensite formed in the welding heat affected zone is liable to cause brittle fracture [3]. The carbon content in the shipbuilding steels that were developed earlier was often as high as 0.3%, and a large amount of manganese was also added with the aim of improving the mechanical properties.

Since the 1950s, NiCrMoV serial shipbuilding steels such as HY80 and HY100 have been successively developed, in which the carbon content was generally controlled to a level lower than 0.18%, and the addition of Ni, Cr and Mo elements contributed to the improvement of hardenability [4]. However, their poor toughness at higher strength levels strictly limited their application. In order to improve the toughness of NiCrMoV steels, some modified heat-treatment techniques or the chemical composition optimization for NiCrMoV steels have been carried out [5,6,7,8,9,10]. For instance, the increasing of the tempering temperature and/or holding time had a significant effect on the morphology of carbide precipitates and subsequently resulted in the decrease in strength but the increase in ductility [6,7]. More recently, quenching and subsequent tempering at different temperatures has been commonly applied as a typical heat-treatment process for NiCrMoV steels, which usually results in a tempered martensite microstructure and then leads to the achievement of an excellent combination of strength and toughness. In particular, a high amount of copper was added, and the ε-Cu precipitates and their strengthening effect were used to enhance the mechanical properties of these kinds of steels [8]. This provides a novel idea for developing shipbuilding steels with good comprehensive properties and excellent weldability, that is, reducing the carbon content, or even adopting ultra-low carbon design to improve the steel weldability, while adding Cu to achieve ε-Cu precipitates and provide an effective remedy for the strength loss caused by the reduction in carbon content.

It has been reported that when the addition of the Cu element was higher than 0.6%, the supersaturated Cu in steel would result in the precipitation of ε-Cu particles during the aging treatment, which could then significantly improve the strength of steel [10]. For example, the addition of an amount of Cu reaching a value of 1% can lead to an increase of about 248 MPa in strength [11,12]. Additionally, Wang et al. [13] found that the ductile-brittle transition temperature of Cu-bearing steel could be decreased to a value below −80 °C after aging at 700 °C and being held for 1 h, indicating that the addition of Cu can not only improve the strength, but also the low-temperature toughness of steel. Recently, the effect of Ti microalloying on the mechanical properties of Cu-bearing steel has also been reported [14]. The results showed that the trace addition of Ti was beneficial for the refinement of nanoscale Cu precipitates, and thereby the improvement of both the strength and toughness of Fe-0.05C-1.3Cu steel. Of course, the heat treatment can also be used to improve the comprehensive properties of Cu-bearing steel. For example, multi-step heat treatment including intercritical treatment and subsequent tempering would reduce Cu segregation and then be effective in improving the toughness, with limited reduction in yield strength [15].

Although some research studies on Cu-bearing age-hardening steel have been reported, its strengthening and toughening mechanism remains unclear due to the absence of comparative studies with traditional NiCrMoV shipbuilding steels. In this paper, an ultra-low-carbon Cu-bearing age-hardening steel developed in our previous studies was subjected to the same heat treatment with NiCrMoV steel, and then the microstructural difference between them was analyzed in detail, with the aim of exploring the effect of microstructural difference on the strength and toughness mechanism.

## 2. Materials and Methods

The hot-rolled sheets of Cu-bearing age-hardening steel and NiCrMoV steel were used in this study. The chemical compositions of the studied steels are listed in Table 1. The thickness of hot-rolled sheets for both steels was 35 mm. A portion of sample with a size of 20 mm × 200 mm × 35 mm was cut directly from the hot-rolled steel sheets and used for the heat treatment.

**Figure 1:**
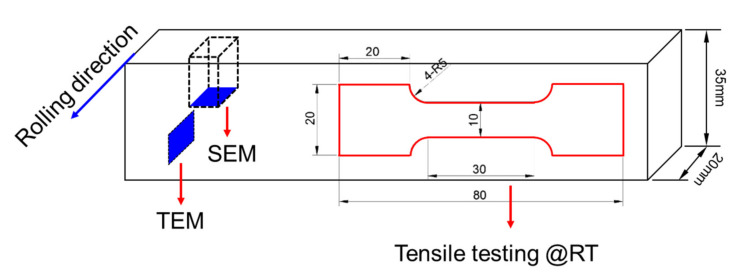
Sample sizes and the schematic diagrams for microstructure observations and tensile tests.

The studied steels were solution-treated for 1 h at 900 °C, followed by water quenching. The quenched slabs were tempered at 630 °C for 2 h and then air-cooled to room temperature. In order to facilitate the comparison of the experimental results, the heat treatment of both Cu-bearing age-hardening steel and NiCrMoV steel were carried out under the same conditions. The samples used for microstructure observations and tensile tests were then machined from the heat-treated slabs by wire-electrode cutting. The thickness of the tensile sample was 2 mm. Detailed size and the schematic diagrams for microstructure observations can be found in Figure 1.

The tensile tests were performed at room temperature on a Zwick/roell Z100 universal testing machine (ZwickRoell Gmbh & Co. KG, Ulm, Germany). The extensometer was used to track the strain to failure. The loading force, displacement and other parameters were recorded by the software during the testing process. The initial rate for the tests was set as 2 mm/min. Three samples for each steel were tested in order to achieve more accurate results.

Standard metallographic procedure was applied prior to the microstructure observation. The surface of all specimens was polished down to 1µm with water-based diamond suspensions and, at the end of the process, cleaning with acetone and drying in air were undertaken. Nital etchant containing 5% nitric acid was used for etching. The EBSD (Electron Back-Scattered Diffraction) samples were prepared by electropolishing with 5% perchloric acid alcohol solution. The voltage was set to 30 V and the polishing time was about 12 s. Ion etching was employed for the preparation of the TEM (Transmission Electron Microscope, FEI Company, Hillsboro, OR, USA) samples with 8% perchloric acid alcohol solution. The microstructure observations via SEM (scanning electron microscope) were carried out on a HITACHI SU5000 (Tokyo, Japan) with an OXFORD HKL (Oxford Instruments plc, Oxfordshire, UK) detector for the EBSD test and EDS (Energy Dispersive Spectroscopy) analysis. As well as an optical microscope (ZEISS Axiovert A1, Carl Zeiss AG, Jena, Germany) equipped with a live camera, the CHANNEL 5 software (Oxford Instruments plc, Oxfordshire, UK) was employed for the EBSD data analysis. The microstructure, tensile fracture and precipitates were further observed using a transmission electron microscope (FEI Tecnai G2 F20, FEI Company, Hillsboro, OR, USA).

## 3. Results and Discussion

### 3.1. Mechanical Properties and Microstructure Observations

**Figure 2:**
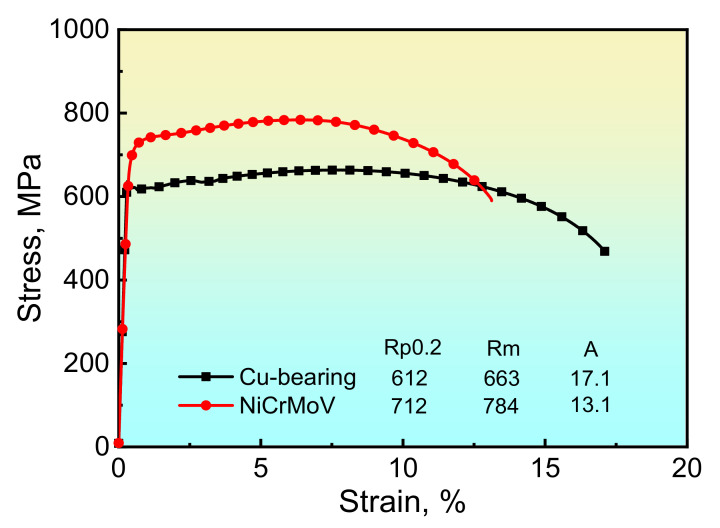
Stress–strain curves of as-tempered samples tensile tested at room temperature.

The engineering stress–strain curves obtained from tensile tests of tempered samples are shown in Figure 2, in which the property data measured from these curves are also illustrated. As can be seen, the yield strength, tensile strength and elongation to fracture of the Cu-bearing age-hardening steel are 612 MPa, 663 MPa and 17.1%, respectively, while the corresponding values for NiCrMoV steel are 712 MPa, 784 MPa and 13.1%, respectively. It is clear that the Cu-bearing age-hardening steel exhibits excellent elongation but a relatively lower strength as compared to NiCrMoV steel.

**Figure 3:**
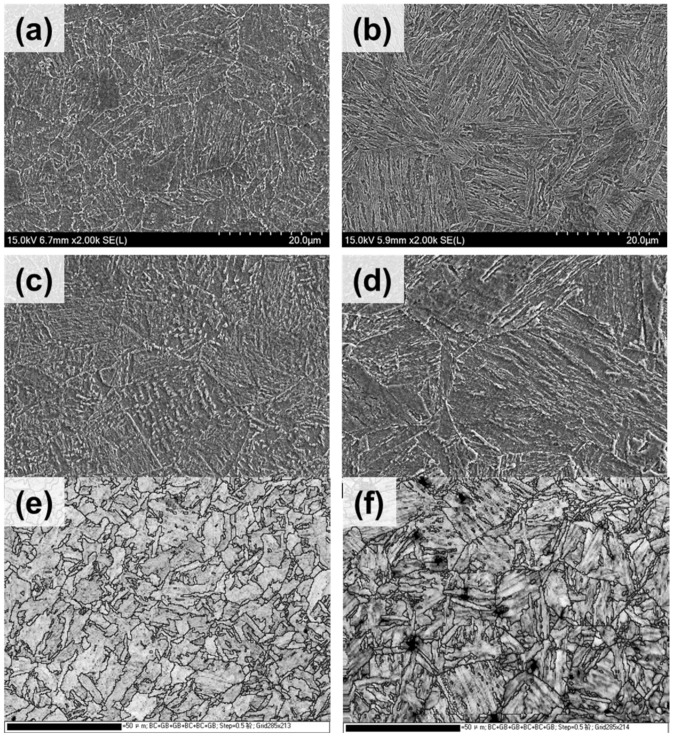
SEM observations (**a**–**d**) and EBSD image quality maps (**e**,**f**) of the studied steels in (**a**,**b**) as-quenched and (**c**–**f**) as-tempered conditions. (**a**,**c**,**d**) Cu-bearing age-hardening steel; (**b**,**d**,**f**) NiCrMoV steel.

Figure 3 shows the SEM and EBSD image quality maps of the quenched and tempered Cu-bearing steel and NiCrMoV steel. The as-quenched microstructures (Figure 3a,b) show that a fully lath-martensitic microstructure can be observed in NiCrMoV steel, while a bainitic microstructure is the primary characteristic of Cu-bearing steel, although the same quenching condition is adopted. The main difference in chemical composition between the steels should be responsible for the microstructure difference. Specially, the relatively higher contents of, for example, Carbon and Chromium, in NiCrMoV steel contribute to the formation of martensite because elements of this type usually have a positive effect in terms of improvements in hardenability.

During tempering, the decomposition of bainite or martensite occurred in both studied steels, which resulted in the achievement of granular bainite in Cu-bearing steel but tempered martensite in NiCrMoV steel (Figure 3c,d). Grain boundaries with misorientations larger than 15° are displayed in EBSD image quality maps (Figure 3e,f), which reveal the prior austenite grains and their distribution before quenching. It can be seen that the substructure characteristic related to the martensite lath still remained in NiCrMoV steel even after tempering, which was responsible for the higher strength but lower elongation to fracture than Cu-bearing steel. However, it is noteworthy that although the matrix microstructure is mainly composed of bainite, and such elements as C, Cr and Ni have a lower addition in Cu-bearing steel, its tensile strength is not much lower than that of NiCrMoV steel.

### 3.2. Precipitates Observation and Analysis

**Figure 4:**
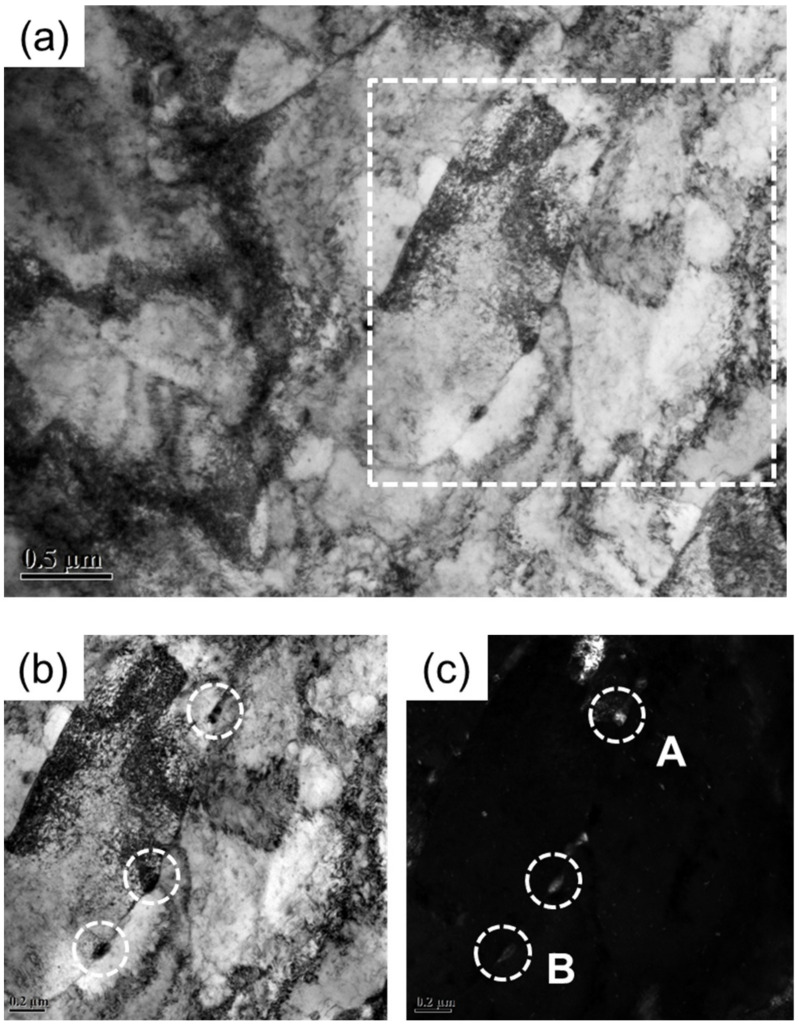
TEM observations of deformed matrix and precipitates in Cu-bearing steel. (**a**) General view of sub-structures; (**b**) bright-field image; (**c**) dark-field image, A and B showing the typical precipitates.

As mentioned above, the addition of Cu, and subsequently ε-Cu, particles precipitated during such heat treatment processes as tempering or aging will significantly improve the strength of Cu-bearing steels [11,12,13]. In order to investigate the effects of Cu on precipitation and mechanical properties, the microstructures in the vicinity of the fracture surface of the tensile tested samples have been examined by TEM analysis. Figure 4 shows TEM images of a tempered Cu-bearing steel after tensile testing at room temperature. As can be seen, some bainite ferrite lath and large amounts of dislocations exist in the bright-field images in Figure 4a. However, due to the tensile deformation at room temperature, the lath boundaries tend to be slightly blurred. This is mainly attributed to the fact that the lath boundaries, similarly to the general grain boundaries, can also retard dislocation movement during the deformation and then result in abundant dislocation pile ups at lath interfaces. This also reveals that the existence of bainite ferrite laths is one of the possible reasons for the improvement of Cu-bearing steel regardless of its lower element contents and bainite microstructure.

**Figure 5:**
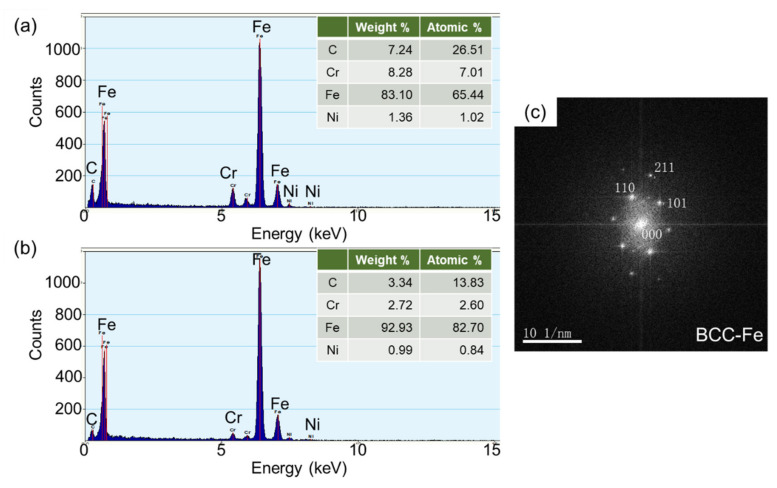
EDS analysis (**a**,**b**) of precipitates A and B in Figure 4c and TEM diffraction spot of particle A (**c**) in Cu-bearing steel.

In addition, the lath boundaries can also serve as nucleation sites for the precipitates during heat treatment. As a result, some fine precipitates (about 150 nm in size) distributed along the bainite ferrite laths can be found in Figure 4b, a zoomed-in bright image of the white dashed area in Figure 4a. The corresponding dark-field image displayed in Figure 4c further verifies the existence of precipitates. In general, it is very difficult for the precipitates formed in the as-quenched steel because the precipitation is usually inhibited by the rapid cooling. However, during tempering or aging, especially at a moderate or elevated temperature, the diffusional decomposition of the supersaturated solid solution such as martensite or bainite will occur, which eventually leads to the precipitation of carbides. In order to qualitatively analyze the type of precipitate particles, EDS was used to determine the chemical composition of the precipitates in Cu-bearing steel. The corresponding results associated with particles at A and B in Figure 4c are shown in Figure 5, in which the dominant elements of both particles A and B are C, Cr, Fe and Ni. Considering the possible types of precipitates formed by each element and the influence of matrix elements in the energy spectrum analysis, it is tentatively inferred that the particles at A and B are Cr-rich carbides. The particles at A were further investigated using high-resolution transmission electron microscopy and the diffraction spot is shown in Figure 5c. Although no clear diffraction spot was obtained during the analysis, it can still be inferred the precipitates are Cr_23_C_6_ by measuring and calculating the interplanar spacing of diffraction spots in combination with EDS analysis.

**Figure 6:**
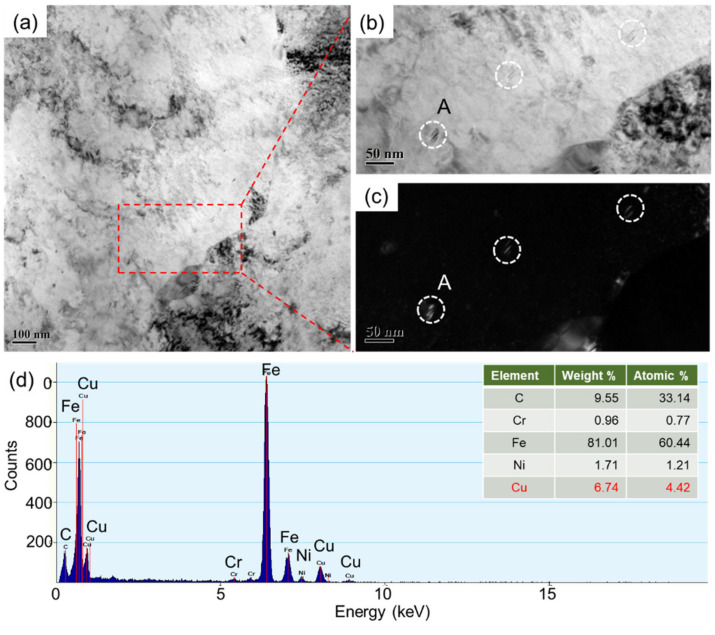
The distribution of Cu-containing precipitates observed by TEM (**a**). The bright field image (**b**) and the dark field image (**c**) shows the morphology and the size of precipitates. The EDS analysis on the precipitate A in (**b**) verifies the Cu-rich in these precipitates (**d**).

It has been reported that ε-Cu or Cu-rich particles will precipitate from the supersaturated matrix when the Cu-bearing steel has been aged at a temperature higher than a certain value [16,17]. As a result, these fine and dispersed particles can induce a significant strengthening effect. In the present study, the Cu-rich particles in the tempered Cu-bearing steel are also found by TEM observations. The morphology, size and distribution of Cu-rich particles are illustrated in Figure 6.

As shown in Figure 6b,c, the size of these Cu-rich particles is smaller compared to Cr carbides, which are about 20–40 nm. Due to their small size, diffraction spots with good quality were not acquired in TEM analysis, but the EDS result in Figure 6d clearly shows a small amount of Cu in the particle composition. Taking into account the dense distribution and small size of Cu-rich phase precipitate particles, they usually have a significant role in the pinning of the dislocations, resulting in significant improvements in the strength of the Cu-bearing steel.

Different from those Cr carbides precipitated along the bainite lath boundaries in Figure 4, the Cu-rich particles are mainly dispersed in the ferrite matrix, mostly in the shape of short rods or ellipsoids, which are very close to the morphology of Cu-rich phase particles as referred to in References [18,19]. The primary reason why the fine Cu-rich phase particles are distributed in ferrite matrix may be attributed to the existence of dislocation and other defects with relatively high energy, which provides some effective transfer channels for Cu diffusion during tempering, as well as appropriate nucleation sites for the precipitation of Cu particles.

In addition, due to the addition of Nb or other alloying elements in the Cu-bearing age-hardening steel, Nb(C,N) particles may also precipitate during the aging of the steel, except during Cu precipitation. It has been reported that the Nb-carbonitride precipitation may also promote the precipitation of Cu particles during aging treatment [20]. NbC precipitation can inhibit the movement of the dislocations and provide more nucleation sites for the precipitation of Cu-rich phase particles, thus increasing the number of precipitated Cu particles. However, it may be possible for the pre-precipitated NbC to induce an increase in the size of Cu particles and the distance between them, and then cause the Cu particles to coarsen, which consequently reduces the strengthening effect of Cu precipitates. Therefore, for the present Cu-bearing steel, the Nb content and the tempering temperature need be strictly controlled, which is an issue that is worthy of further study.

**Figure 7:**
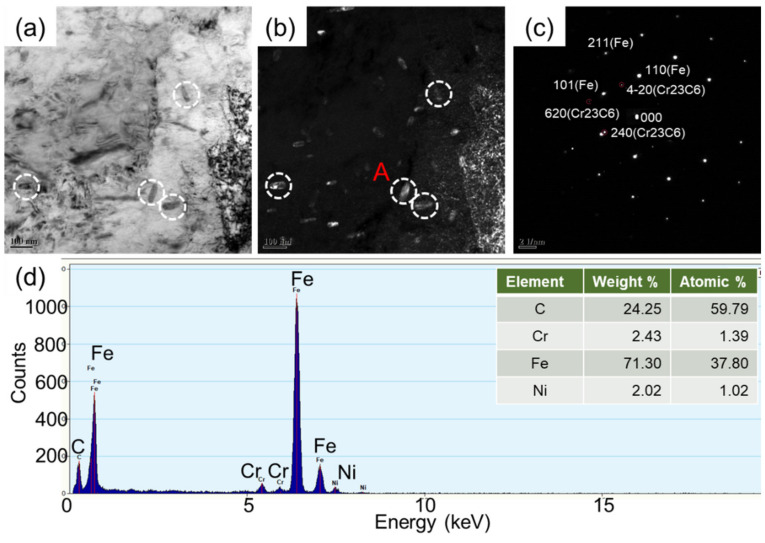
Bright-field (**a**) and dark-field (**b**) images of precipitates in NiCrMoV steel. The corresponding TEM diffraction spot (**c**) and EDS analysis (**d**) of particle A showing the existence of Cr-rich precipitates.

Figure 7 shows the TEM analysis of the matrix and precipitates in the tempered NiCrMoV sample after tensile testing. Similarly to those in Cu-bearing steel (Figure 4), dispersed second-phase particles can also be found in the NiCrMoV steel matrix. As can be seen from the bright and dark field images in Figure 7a,b, the precipitates are generally short rod-shaped, and are about 120 nm in size. In order to further clarify the possible types of precipitates, the EDS energy spectrum was also studied for the purpose of analyzing the composition of the second-phase particles in Figure 7d. The results illustrate that the main alloying elements of the precipitates are Cr, Fe and Ni. Combined with the diffraction spots of the precipitate in Figure 7c, the precipitates may be identified as Cr_23_C_6_ particles.

### 3.3. Comparison of the Mechanism of Strengthening and Toughness

Note that the carbon content of Cu-bearing age-hardening steel and NiCrMoV steel in our present work is 0.037 and 0.077%, respectively. The solid solution strengthening effect resulting from carbon may be negligible. Furthermore, there are only trace additions of Ti or Nb in the studied steels; the precipitation hardening caused by possible precipitates such as Ti or Nb carbonitrides is also not significant. Therefore, the additions of the Ni, Cr and Mo elements play an essential role in the enhancement of the strength of both steels due to their effective improvement of hardenability. This is the primary reason that some martensite or bainite laths can still be found in both steels even after tempering. Nevertheless, the carbon and other alloying elements in Cu-bearing steel are relatively lower than those in NiCrMoV steel, resulting in small amounts of granular bainite appearing in Cu-bearing steel due to the insufficient hardenability.

The TEM analysis in Figure 4 and Figure 6 demonstrates that two types of precipitates, Cr carbides and Cu-rich particles, with totally different morphologies and distributions, are formed in Cu-bearing age-hardening steel after tempering. The precipitated Cr carbide particles are relatively large in size, and mostly distributed along the lath boundaries. These Cr carbides can delay the decomposition of martensite or bainite lath during tempering, and also play the role of reinforcing the lath during plastic deformation. 

**Figure 8:**
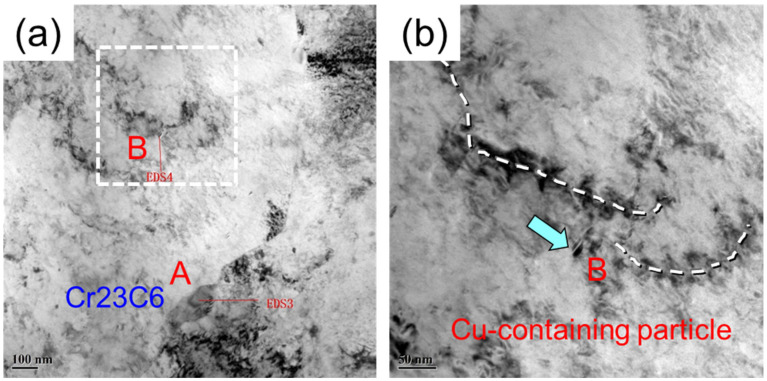
The dislocation pile-up and multiplication around the precipitates during the tensile test of Cu-bearing age-hardening steel. (**a**) interaction between precipitates and dislocations; (**b**) a zoomed-in image of the white dashed area in (**a**), showing the dislocation pile-up and multiplication resulted from Cu-rich precipitates.

Although Cr carbides have been found in both the tested steels, with particle sizes of about 120–150 nm, finer Cu-rich phase particles of about 20–40 nm in size are only present in the Cu-bearing age-hardening steel. Furthermore, Cu-rich phase particles are relatively small in size and mainly distributed in the ferrite matrix. During plastic deformation, these particles play the role of pinning the dislocations and other defects, which is verified by the TEM observations in Figure 8. It can be seen that the dislocation density near these precipitates is usually much higher than that in the surrounding microstructure (Figure 8a); specifically, the precipitates hinder the dislocation movement during tensile deformation, resulting in dislocation pile-ups and even contributing to dislocation multiplication (Figure 8b).

The presence of second-phase particles exerts a certain influence on the strength and plasticity of material. It is generally believed that the strength of a material can be effectively improved when fine second-phase particles are dispersed in the matrix. Moreover, the research results for Nano steel show that a reasonable size and distribution of second-phase particles can also enhance the plasticity and toughness of steels at the same time [21,22,23]. Therefore, the interactions of Cr carbides and Cu-rich phase particles with dislocations and laths, as well as the strengthening effect arising from the interactions, make up for the strength loss caused by the decrease in carbon content, and make a great contribution to the improvement of the strength of Cu-bearing age-hardening steel. 

## 4. Conclusions

The study was focused on the differences between Cu-bearing age-hardening steel and NiCrMoV steel in terms of their strengthening and toughening mechanisms. It can be concluded from the experimental study and analysis that:

1. As compared to conventional NiCrMoV steel, the Cu-bearing age-hardening steel exhibits a relatively poor hardenability. After quenching and subsequent tempering, the microstructure of Cu-bearing age-hardening steel consists largely of bainite.
2. Cr carbides have been found in both tested steels, with particle sizes of about 120–150 nm. However, some finer Cu-rich phase particles of about 20–40 nm in size can also been found in the Cu-bearing age-hardening steel.
3. Cu plays a leading role in precipitation hardening, making up for the strength loss in Cu-bearing age-hardening steel due to the content reduction of carbon and other alloying elements. The interactions between Cu particles and dislocations result in dislocation multiplication, which contributes to the better plasticity of Cu-bearing age-hardening steel.

## Figures and Tables

**Table 1:** Chemical compositions of the Cu-bearing age-hardening steel and NiCrMoV steel (mass, %).

Steel	C	Si	Mn	P	S	Ni	Cr	Mo	V	Cu	Nb	Ti	Als
Cu-bearing	0.037	0.27	0.58	0.007	0.002	1.92	0.78	0.24	-	1.36	0.019	0.014	0.026
NiCroMoV	0.077	0.21	0.42	0.011	0.005	2.74	1.00	0.22	0.06	-	-	0.015	0.04

## Data Availability

The data presented in this study are available on request from the corresponding author.
